# Extrahypothalamic Control of Energy Balance and Its Connection with Reproduction: Roles of the Amygdala

**DOI:** 10.3390/metabo11120837

**Published:** 2021-12-03

**Authors:** Rafael Pineda, Encarnacion Torres, Manuel Tena-Sempere

**Affiliations:** 1 Instituto Maimónides de Investigación Biomédica de Cordoba (IMIBIC), 14004 Cordoba, Spain; bo2tojie@uco.es; 2 Department of Cell Biology, Physiology and Immunology, University of Cordoba, 14004 Cordoba, Spain; 3 Hospital Universitario Reina Sofia, 14004 Cordoba, Spain; 4 CIBER Fisiopatología de la Obesidad y Nutrición, Instituto de Salud Carlos III, 14004 Cordoba, Spain; 5 Institute of Biomedicine, University of Turku, FIN-20520 Turku, Finland

**Keywords:** metabolism, amygdala, estrogens, neuropeptides, kisspeptins, food intake, energy balance, body weight

## Abstract

Body energy and metabolic homeostasis are exquisitely controlled by multiple, often overlapping regulatory mechanisms, which permit the tight adjustment between fuel reserves, internal needs, and environmental (e.g., nutritional) conditions. As such, this function is sensitive to and closely connected with other relevant bodily systems, including reproduction and gonadal function. The aim of this mini-review article is to summarize the most salient experimental data supporting a role of the amygdala as a key brain region for emotional learning and behavior, including reward processing, in the physiological control of feeding and energy balance. In particular, a major focus will be placed on the putative interplay between reproductive signals and amygdala pathways, as it pertains to the control of metabolism, as complementary, extrahypothalamic circuit for the integral control of energy balance and gonadal function.

## 1. Introduction: The Amygdala

The amygdala, also known as amygdaloid complex (AC), is a brain region that in mammals comprises several nuclei or groups of nuclei, distinguished and labelled on the basis of their cytoarchitecture, histochemistry, and inter-connections. In rodents, these different nuclei are divided into three main groups: (i) the deep or basolateral group, which includes the lateral, basal, and accessory basal nuclei; (ii) the superficial or cortical group, which includes the cortical nuclei and nucleus of the lateral olfactory tract; and (iii) the centromedial group, which includes the medial and central nuclei. In addition, other accessory nuclei, including the intercalated cell masses and the amygdalo-hippocampal area, have been also described [1,2,3].

The amygdala has been the focus of active research in different domains of neurosciences and neuroendocrinology since its discovery in the early 19th century. While exhaustive recapitulation of the physiological roles of AC as a key center for emotional learning and behavior, is beyond the scope of this review and can be found elsewhere [1,2,3]; for the interest of this work, it is important to stress that compelling evidence highlights that the amygdala is involved in the control several behaviors related to feeding, such as food intake, appetitive conditioning, gustatory neophobia, taste aversion, and food conditional-place preference. Thus, the AC seemingly contributes in a dual manner to the control of homeostatic and hedonic/reward eating [4,5,6]; while the former is driven mainly by energy needs, the latter is not directed primarily to satisfy energy demands, but rather responds to hedonic cues, and hence can favor overweightness [7,8]. This has drawn considerable attention and research interest, given the escalating prevalence of obesity and its severe disease burden in human health.

In fact, initial studies mapping the physiological roles of different amygdala areas, based on chemical or physical lesions of specific regions of the AC, have already highlighted the connection of amygdala with feeding control. This is epitomized by the seminal work of King and co-workers, showing that amygdaloid lesions, e.g., in the posterodorsal amygdala, lead to hyperphagia and weight gain in male and female rats [9,10,11]. These studies not only documented a role of AC in feeding control, but also in the regulation of other key metabolic parameters, such as glycemia, and supported the role of amygdala not only in the homeostatic control of body weight, but also in food preferences and selection of macronutrients [11]. Admittedly, however, while this work paved the way for elucidation of the role of amygdala in the control of feeding and energy homeostasis, it suffered from important technical limitations, mainly related to the lack of precise resolution and the inability to discriminate the effects derived from the lesion of nuclei located in the regions damaged or of projections passing through them. Hence, more sophisticated approaches, involving neuronal tracing, functional genomics, and viral/pharmacogenetics, have been implemented to tease apart the roles of specific amygdala pathways in the control of metabolic homeostasis. As relevant recent example, elegant neurophysiological and functional studies in mice have documented that a GABAergic neural pathway, expressing type 2a serotonin receptors, located in the central nucleus of the amygdala (CeA) plays a key role in the control of food consumption, in close connection with other brain regions involved in feeding control [12].

## 2. A Tight Connection: The Link between Energy Homeostasis and Reproductive Function

Reproduction is an essential function for perpetuation of the species and, hence, its control is subjected, as is also the case of feeding, to sophisticated regulatory mechanisms to ensure, whenever feasible, maximal reproductive efficiency. However, fertility is dispensable at the individual level, and, considering its high metabolic and energy demands, it makes biological sense that it can be only achieved or maintained when sufficient body energy reserves are attained to afford its metabolic drainage, especially in the female (i.e., during pregnancy and lactation). Hence, situations of body energy deficit and/or metabolic distress, ranging from anorexia to severe obesity, are often associated with pubertal perturbations and sub/infertility [13]. Nevertheless, the suppressive effect of adverse metabolic conditions on the reproductive axis, as a means to minimize energy disposal and maximize reproductive efficiency, must be controlled accurately, to ensure resuming of fertility as soon as a more favorable metabolic status is achieved. Importantly, not only metabolic cues modulate reproductive function, but, conversely, gonadal signals are also important metabolic modulators, with capacity to control key aspects of energy homeostasis [14].

While detailed recapitulation of the mechanisms whereby metabolism and reproduction are tightly connected exceeds the scope of this review and can be found elsewhere [13,15], for the objectives of this review it is important to stress that this complex physiological phenomenon relies on an array of regulatory networks, involving different hypothalamic and extra-hypothalamic circuits, as well as numerous peripheral factors, that adjust reproductive maturation and function to the endogenous metabolic conditions and environmental cues. These include, prominently, brain pathways controlling both reproduction and metabolism, as well as metabolic and gonadal hormones, which impinge upon the so-called hypothalamic–pituitary–gonadal (HPG) axis. In this axis, hypothalamic neurons that synthesize the decapeptide, gonadotropin-releasing hormone (GnRH), are a major hierarchical component, as they act as final integrators of different central and peripheral signals, and major output pathway for the brain control of the downstream elements of the HPG axis [16,17]. Of note, GnRH neurons appear to be devoid of key metabolic sensors, suggesting that afferents to GnRH neurons are responsible for sensing and transmitting the modulatory effects of different metabolic signals [13,17].

Importantly, while the metabolic gating of female reproduction is intuitively explained by the considerable metabolic demands of pregnancy and lactation, it must be stressed that key aspects of reproductive behaviors, affecting prominently males, such as aggression, mating, territoriality, and dominance, also depend on sufficient energy reserves [15]. Thus, metabolic control of reproduction, in an ample sense, does not only depend on mechanisms controlling the hormonal reproductive axis, but also on pathways controlling key behaviors. In this context, the amygdala has emerged as relevant brain area for the regulation of key metabolic and reproductive neuroendocrine functions and behaviors.

In this mini-review, we will focus our attention on three elements that may participate in such integral regulatory mechanisms, also engaging the amygdala, which include: (i) metabolic neuro-peptide pathways; (ii) the kisspeptin system; and (iii) gonadal hormones. These will be reviewed in the following sections. As a search method, we have implemented a comprehensive MEDLINE search, using PubMed as main interface, of research articles and reviews, published mainly between 2005 and 2021, using previously published guidelines [18]. In detail, search was implemented using multiple keywords, including amygdala, metabolism, estrogens, androgens, reproduction, neuropeptides, kisspeptin, food intake, energy balance, and body weight, with the Boolean operators AND/OR, focusing mainly on preclinical data connecting amygdala, energy balance, and reproductive function. In addition, studies addressing the role of the amygdala in the physiological control of feeding and energy balance were also considered, and, when relevant, the web application Connected Papers (https://www.connectedpapers.com/, accessed on 15 November 2021) was used to comprehensively cover all key references in specific topics of the review.

## 3. Metabolic Neuropeptide Pathways and the Amygdala: Roles of NPY/AgRP and POMC

A major circuit for the homeostatic control of body weight and energy balance is placed in the hypothalamic arcuate nucleus (ARC), and involves the reciprocal interplay of two populations of neurons, with opposite roles in the control of feeding, namely neurons expressing proopiomelanocortin (aka, POMC neurons, which conduct anorexigenic actions) and neurons expressing neuropeptide Y/agouti-related peptide (aka, NPY/AgRP neurons, with dominant orexigenic actions) [19,20,21]. In a broader perspective, POMC and NPY/AgRP neurons have been also shown to participate in the modulation of the reproductive axis, and may contribute to the integral control of reproduction and metabolism [13]. In this section, we will briefly summarize available evidence supporting a role of these neuropeptide pathways in the control of amygdala and related functions.

Anorexigenic POMC neurons in the ARC have been defined as critical in the control of body weight and energy homeostasis [22]. Of note, a second population of POMC neurons has been found in the nucleus of solitary tract (NTS), but its role controlling energy homeostasis seems less relevant than that of the ARC population [23]. ARC POMC neurons express a panoply of neuropeptides, such as melanocortins, β-endorphin, and cocaine and amphetamine-regulated transcript (CART), as well as γ-aminobutyric acid (GABA) and glutamate neurotransmitters [22,24]. In addition to adreno-corticotropic hormone (ACTH) produced in the anterior pituitary, melanocortin peptides include α-, β-, and γ-melanocyte-stimulating hormones (MSH), derived from post-translational processing of POMC [22], which are all involved in the control of energy homeostasis [25]. Nevertheless, the major ARC POMC neuronal product is α-MSH, which operates via two of the five melanocortin receptor (MCR) subtypes, namely MC3R and MC4R. These MCR are expressed in the hypothalamus, as well as in other brain regions [26].

**Figure 1:**
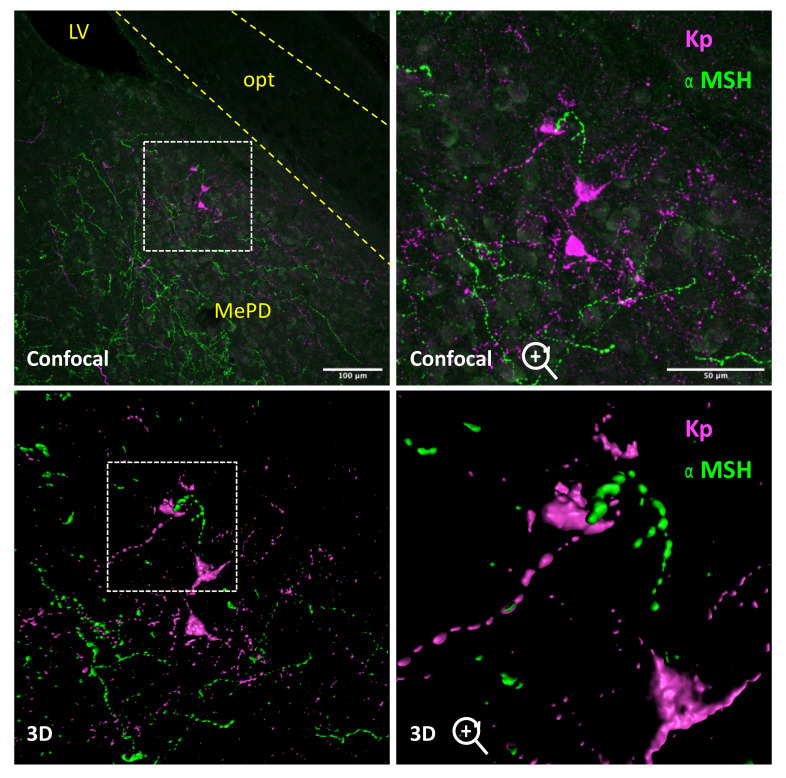
Rat amygdala Kiss1 neurons receive melanocortin inputs. Confocal images and 3D reconstructions of amygdala Kiss1 neurons (magenta) receiving α-MSH (green) appositions. Coronal section of an adult male rat at bregma level—3.60 mm. Materials (antibodies) and methods are described in detail elsewhere [32]. LV = Lateral ventricle; opt = Optic track; MePD = Postero-dorsal area of the medial amygdala (MeA).

Admittedly, the connection of POMC signaling pathways and the amygdala remains unfolded, but fragmentary evidence suggests a potential bidirectional interplay between POMC neurons and amygdala circuits. Thus, MC4R has been shown to be highly expressed in the amygdala [27], particularly in the MeA [28]. Moreover, central infusion of the MC3R/MC4R agonist, melanotan II, into the CeA caused a marked and long-lasting decrease of food intake in rats, in a dose dependent-manner; responses that were higher in animals fed a high-fat diet (HFD). Conversely, injection of the MCR antagonist, SHU-9119, caused hyperphagia [29]. These pharmacological data argue in favor of a role of direct α-MSH effects in the amygdala to modulate feeding. This contention has been recently documented by elegant studies from Kwon and Jo, showing by a combination of molecular tracing and optogenetic experiments, that a circuit originating from ARC POMC neurons projects to neurons in the medial amygdala (MeA), which express not only MC4R, but also estrogen receptors, whose activation reduces food intake, in a MC4R-dependent manner [30]. In the same vein, we present herein our previously unpublished evidence in the rat for the presence of α-MSH immunoreactive fibers surrounding/in close contact with another neuronal population in the amygdala, namely Kiss1 neurons (Figure 1). While this Kiss1 neuronal population will be described in detail in Section 4 of this review, it is interesting to note that other subpopulations of Kiss1 neurons, in the rostral hypothalamus, have been shown to express MC4R [31]; whether the same applies to amygdala Kiss1 cells awaits future investigation.

In addition to the projections of ARC POMC neurons to the amygdala, it has been demonstrated that POMC neurons in the NTS receive projections from the amygdala [33]; this population of NTS POMC neurons participates in the short-term inhibitory control of feeding [34], in contrast to the long-term anorexigenic actions of ARC POMC neurons. In fact, our unpublished data show that acute fasting in rats suppresses *POMC* expression more potently in the NTS than in the ARC (Pineda and Torres, unpublished), supporting a major role of POMC neurons in the brain stem in the acute control of feeding. The fact that this population receives projections from the amygdala strongly suggests that this pathway might contribute to the role of amygdala in the modulation of feeding responses in conditions of acute metabolic stress [35].

As counterbalance to the anorectic actions of melanocortins, NPY is a highly conserved, widely distributed neuropeptide, one of the most abundantly expressed in the mammalian brain, which conducts strong orexigenic actions, acting mainly via two subtypes of the Y receptors, namely Y1 and Y5 [36,37]. Neurons expressing NPY are found in several brain areas, including prominently the ARC, and also the amygdala [38]. Of note, ARC NPY cells co-express AgRP, which acts as functional antagonist of MC3R and MC4R [39], thereby also driving a potent orexigenic effect. Accordingly, NPY/AgRP expression markedly increases in the hypothalamus under conditions of food deprivation [40]. In the rat, NPY neurons are also found in the AC, especially in the medial and lateral amygdaloid nuclei [41]; expression of NPY receptors has been also reported in the centromedial amygdala [38]. Of note, amygdala NPY neurons are found in roughly the same coordinates/region as amygdaloid Kiss1 neurons [42]; for further details see Section 4. However, co-expression of these two neuropeptides in the same amygdala cells and/or their interactions have not been documented yet. Similarly, whether amygdala NPY expression is modulated under conditions of nutritional stress (e.g., fasting) is yet to be clarified. Interestingly, intra-amygdala injection of NPY (in the CeA) has been shown to alter food preference and macronutrient selection in fed and overnight fasted rats, decreasing preference for a high-fat content diet, but without changing total calorie intake [43]. These data suggest that amygdaloid NPY signaling may play specific roles in feeding control, beyond the orexigenic, energy homeostatic actions of ARC NPY. In this context, very recent studies have highlighted that the amygdala population of NPY neurons originating in the CeA may play a relevant role in promoting feeding, specifically under conditions of chronic stress. Thus, selective over-expression of NPY in the CeA led to increased feeding and decreased energy expenditure, thereby promoting an obesity phenotype, when chronic stress and high fat diet were combined [44]. Again, these findings would argue for specific roles of NPY signaling originating from the amygdala in the control of feeding and energy balance.

Less is known about the putative roles of AgRP signaling in the control of AC, although ARC AgRP neurons have been shown to project to several extrahypothalamic areas, including the CeA. Of note, however, the physiological roles of these amygdala projections are yet to be elucidated, as specific activation of this pathway was insufficient, per se, to evoke feeding [45].

Notably, while there is ample consensus that peripheral metabolic hormones operate primarily on hypothalamic circuits to convey their regulatory actions on feeding and energy homeostasis, fragmentary evidence suggests that amygdala circuits might be also modulated (directly or indirectly) by key metabolic hormones to conduct at least part of their regulatory actions. This is the case of insulin, a key metabolic hormone with potent anorectic effects acting at central levels. Very recent evidence has documented that suppression of insulin signaling on CeA NPY neurons is a major mechanism for the development of hyperphagia and obesity in a mouse model of HFD and chronic stress [44]. In addition, loss of insulin signaling in the CeA has been shown to decrease core body temperature through direct regulation of brown adipose tissue activity [46], thereby modulating cold-induced thermogenesis and energy balance.

As final note in this section, it must be stressed that both POMC and NPY/ AgRP neurons in the hypothalamus have been reported to participate in the control of the reproductive axis [13,32,47]. However, whether the amygdala circuits involving these neuropeptides actually contribute to such regulatory function is yet to be fully clarified and warrants future investigation. For additional comments on this issue, see Section 4.

## 4. The Kisspeptin System and the Amygdala

While different neuropeptide pathways other than NPY/AgRP and POMC have been found in the amygdala, with potential connections with the control of metabolism and/or reproduction, the recent discovery of the expression and putative functions of the Kiss1 system in the amygdala has drawn considerable interest, as this might illuminate the underlying circuits connecting different essential behaviors closely related with reproduction and, eventually, metabolic homeostasis.

Kisspeptins are a family of structurally related peptides with a distinctive RF-amide motif at the C-terminus, encoded by the Kiss1 gene, that operate via the G protein-coupled receptor Kiss1R (also known as Gpr54). Discovery of the reproductive dimension of kisspeptins in late 2003 is now considered a major breakthrough in reproductive endocrinology, as kisspeptins play central roles in virtually all major aspects of reproductive maturation and function, from puberty onset to adult fertility. While the physiology of kisspeptins has been extensively reviewed elsewhere [18,48,49], for the purpose of this review, it is important to stress that the Kiss1 system has been shown to play crucial roles in the metabolic control of the reproductive axis [50], and may participate in the direct modulation of different aspects of metabolism, from body weight to glucose homeostasis and thermogenesis [51,52], whose physiological relevance is yet to be fully defined.

In rodents, two major populations of Kiss1 neurons are found in the ARC and the anteroventral periventricular nuclei (AVPV) of the hypothalamus. These are sensitive to sex steroid hormones, and play fundamental roles in the control of the pulsatile and surge modes of secretion of GnRH and gonadotropins by mediating the negative and positive (this is in females only) feedback actions of gonadal steroids [53]. Notably, a third population of Kiss1-expressing neurons has been identified in the amygdala, particularly in the MeA [31,42,54,55,56,57,58,59]. As it is the case for hypothalamic Kiss1 neurons, this amygdala Kiss1 neuronal population is also sexually dimorphic, but contrary to the AVPV, amygdala Kiss1 expression is higher in males [57], with null or negligible expression in early post-natal periods in rats and mice [58,60]. These data suggest that, in contrast to the hypothalamus, the Kiss1 neuronal population in the amygdala arises during puberty, possibly driven by the escalating levels of circulating sex steroids coming from maturing gonads.

The neuroanatomy of the Kiss1 neuronal population in the amygdala has begun to be elucidated in recent years. Thus, in situ hybridization and immunohistochemical studies have documented that Kiss1 neurons at this site are located at the postero-dorsal domain of MeA [42,57]. By a combination of tracing techniques, it has been shown that this population of Kiss1 neurons receive appositions from vasopressin and dopaminergic neurons, and display reciprocal connectivity with the accessory olfactory bulb [42]. Moreover, Kiss1 neurons in the MeA also project to GnRH neurons, a pathway that may contribute to the modulation of the gonadotropic axis by environmental cues, such as odor stimuli [42]. In addition, we have found close contacts of MeA Kiss1 neurons and α-MSH fibers in rats (see Figure 1), whose physiological role is yet to be defined.

Accumulating evidence from functional studies has pointed out that the amygdala population of Kiss1 neurons does play a role in the control of the reproductive axis. Thus, while peripheral administration of kisspeptin decreased neuronal activity in the amygdala, intra-amygdala injection of kisspeptin, at the MeA, induced LH secretion [61], whereas intra-MeA infusion of a kisspeptin antagonist reduced LH secretion and pulse frequency in rodents [61]. In good agreement, optogenetic activation of Kiss1 neurons in the MeA resulted in increased LH pulsatility in female mice [62]. Similarly, chemogenetic activation of Kiss1 neurons in the postero-dorsal medial amygdala has been also shown to induce LH secretion [63]. In any event, the roles of amygdala kisspeptin signaling are not restricted to the control of gonadotropin secretion in adulthood, and may involve the modulation of pubertal timing [64], and, more importantly, relevant sex behaviors [65], including partner preference and pheromonal responses [66,67]. While fragmentary evidence has suggested that kisspeptins (or Kiss1 neurons) may modulate feeding in rodents [51], whether this particular amygdala Kiss1 pathway participates in the control of feeding behavior has not been addressed to date.

Interestingly, amygdala Kiss1 neurons are sensitive to the sex steroid milieu, and both estradiol and testosterone (possibly via aromatization to estrogen) upregulates Kiss1 mRNA expression in the MeA [57], an effect that is conducted via estrogen receptor-⍺ (ER⍺), but not ERβ [68]. In fact, a recent report has documented that a large proportion of Kiss1 neurons in the MeA co-express ER⍺ [69], therefore providing the basis for the estrogenic regulation of this neuronal circuit. Of note, as will be described in detail in the following section, targeted ablation of ER⍺ in the amygdala using the SIM1-Cre mouse revealed a role of estrogen signaling at this site in the modulation of body weight, so that mice with selective ablation of ER⍺ in SIM1 neurons displayed an obesity phenotype [70]. Considering that SIM1- and Kiss1 neurons in the amygdala share a similar neuroanatomical location, and both populations abundantly express ER⍺, it remains plausible that the above genetic approach might have also caused ablation of ER⍺ in amygdala Kiss1 neurons, which may, thereby, contribute to mediate at least part of the effects of estrogen, acting at this site, on body weight homeostasis. However, this intriguing possibility is yet to be experimentally tested. Overall, while the evidence suggesting a role of amygdala Kiss1 neurons in the control of reproductive hormones and behaviors is solid, whether this pathway participates also in the bidirectional connection between metabolism and reproduction remains largely unexplored, and requires further investigation.

## 5. Sex Steroids and the Amygdala: Roles of Estrogens and Androgens

Sex steroids, as major products of the gonads, are not only essential players in the control of the neuroendocrine axis governing reproduction, but are also relevant modulators of key aspects of metabolic homeostasis, from food intake to thermogenesis [14]. While a substantial fraction of these metabolic actions are conducted at the level of the hypothalamus [14], we will review the evidence supporting a role of sex steroids in the amygdala, as putative mechanism for the control of body weight and energy balance.

Estrogens exist in three major forms: estrone (E1), 17β-estradiol (E2), and estriol (E3). These exert their effects through three main receptors: the classical ER⍺, ERβ, and the G protein-coupled receptor, GPER/GPR30 [71]. Besides their well-known effects in the control of reproductive function, estrogens are relevant elements in metabolic regulation, with prominent roles in the control of feeding (with anorexigenic effects) and energy expenditure (increasing thermogenesis and energy consumption) [14,72]. The effects of estrogens on energy homeostasis are mainly conveyed via ER⍺, as genetic deletion of this receptor subtype blunts the effects of estradiol on feeding and body weight [73]. In good agreement, women with polymorphisms in the ER⍺ gene [74], or men with genetic inactivation of ER⍺ [75,76], suffer increased adiposity. A substantial component of such metabolic effects of ER⍺ signaling are conducted in the brain, as demonstrated by studies involving selective deletion of this receptor in populations of hypothalamic neurons [77].

The first evidence suggesting the contribution of estrogen signaling in the amygdala to energy homeostasis came from classical experiments showing that placement of implants of estradiol benzoate into the amygdala suppressed food intake in female rats [78]. In the same vein, it has been shown that estradiol modulates the neuronal activation induced by feeding, as measured by c-Fos, in different brain areas, including the CeA [79]. Functional genomic approaches have further refined these observations. As mentioned in the previous section, seminal studies by Xu and co-workers demonstrated that selective congenital removal of ER⍺ from SIM1 neurons, which are mainly found in the MeA and co-express ER⍺ (>40% of them), increased body weight gain [70]. Moreover, when ER⍺ was selectively deleted in the MeA of adult male mice, using an adeno-associated virus approach, the same phenotype was produced [70]. However, the obesity-inducing impact of ablation of ER⍺ signaling from the amygdala was not apparently related with changes in feeding, but rather with a decrease in physical activity, which enhanced the susceptibility to develop obesity in both sexes, and further increased body weight gain after exposure to HFD [70]. In good agreement, over-expression of ER⍺ in the MeA reduced the obesity phenotype, while pharmacogenetic activation of MeA SIM1 neurons increased physical activity [70]. Future work involving chemo- or optogenetic activation/inactivation of SIM1 vs. ER⍺-expressing neurons in the amygdala will help to further delineate the physiological role of this pathway in the control of body weight and energy balance.

In addition to estrogens, androgens are also known to modulate energy balance. Yet, in contrast to estrogens, androgens have been shown to stimulate feeding in several species [80,81,82,83,84]. Of note, brain effects of testosterone, as the main male sex steroid, are largely conducted via conversion to estradiol, in a region and cell-specific manner, by the action of the enzyme, aromatase, encoded by the CYP19 gene [85]. While the major actions of androgens in the central control of metabolism are thought to be conducted at the hypothalamus, high levels of androgen receptor (AR) expression have been reported in the MeA, with higher levels in males than in females [86,87]. In addition, expression of aromatase is also found in the MeA [88,89], therefore providing the basis for local conversion of androgens into estrogens, which, in turn, might further influence body energy balance. However, whether androgen signaling in the amygdala may contribute to regulation of energy homeostasis remains unexplored.

## 6. Summary and Conclusions

Compelling evidence has demonstrated that the amygdala, as a key brain area involved in emotional learning and behavioral control, including reward processing, plays a salient role in the regulation of various aspects of feeding behavior [19], a phenomenon also observed in humans, especially as it pertains to food choices and hedonic decisions [90]. As direct consequence, the amygdala contributes also to maintaining body energy balance, and possibly physical activity, and participates in responses to different forms of metabolic stress, ranging from starvation to obesity. This function is seemingly conducted, at least partially, by the interplay with metabolic neuropeptide systems, with key roles in energy homeostasis, such as POMC and NPY/AgRP, and is modulated by sex steroids, prominently estrogens. Given the proven roles of these signals in the control of reproductive function, and the known interplay between gonadal function and metabolism, it is tenable to propose that these amygdala circuits, as well as other related pathways, such as possibly amygdala Kiss1 neurons, might also contribute to the integral control of reproduction and metabolism. Admittedly, however, most of the data suggesting such a role are indirect or circumstantial, and hence, further research is needed to fully characterize the actual roles of amygdala circuits in defining these behaviors (i.e., feeding, reproductive) which are essential for survival, and the interplay between them.
